# Stubborn Exercise Responders–Where to Next?

**DOI:** 10.3390/sports10060095

**Published:** 2022-06-10

**Authors:** Leo R. Bell, Tim J. Gabbett, Gregory M. Davis, Matthew P. Wallen, Brendan J. O’Brien

**Affiliations:** 1 Institute of Health and Wellbeing, Federation University, Mount Helen, VIC 3350, Australia; lr.bell@federation.edu.au (L.R.B.); tim@gabbettperformance.com.au (T.J.G.); greg.davis@federation.edu.au (G.M.D.); matthew.wallen@flinders.edu.au (M.P.W.); 2 Gabbett Performance Solutions, Brisbane, QLD 4011, Australia; 3 Caring Futures Institute, College of Nursing and Health Sciences, Flinders University, Adelaide, SA 5000, Australia

**Keywords:** exercise, responders, poor, low, training, biomarkers

## Abstract

There is a wide variance in the magnitude of physiological adaptations after resistance or endurance training. The incidence of “non” or “poor” responders to training has been reported to represent as high as 40% of the project’s sample. However, the incidence of poor responders to training can be ameliorated with manipulation of either the training frequency, intensity, type and duration. Additionally, global non-response to cardio-respiratory fitness training is eliminated when evaluating several health measures beyond just the target variables as at least one or more measure improves. More research is required to determine if altering resistance training variables results in a more favourable response in individuals with an initial poor response to resistance training. Moreover, we recommend abandoning the term “poor” responders, as ultimately the magnitude of change in cardiorespiratory fitness in response to endurance training is similar in “poor” and “high” responders if the training frequency is subsequently increased. Therefore, we propose “stubborn” responders as a more appropriate term. Future research should focus on developing viable physiological and lifestyle screening tests that identify likely stubborn responders to conventional exercise training guidelines before the individual engages with training. Exerkines, DNA damage, metabolomic responses in blood, saliva and breath, gene sequence, gene expression and epigenetics are candidate biomarkers that warrant investigation into their relationship with trainability. Crucially, viable biomarker screening tests should show good construct validity to distinguish between different exercise loads, and possess excellent sensitivity and reliability. Furthermore “red flag” tests of likely poor responders to training should be practical to assess in clinical settings and be affordable and non-invasive. Early identification of stubborn responders would enable optimization of training programs from the onset of training to maintain exercise motivation and optimize the impact on training adaptations and health.

## 1. Introduction

Physical activity guidelines recommend that adults regularly participate in endurance and muscle-strengthening exercises [1]. Whilst prolonged sitting time and sedentary behaviour increase the risk of all-cause mortality, 60 to 75 min of at least moderate-intensity exercise per day appears sufficient to attenuate this risk [2]. Regular participation in endurance exercise increases cardiorespiratory fitness [3] and lowers the risk of chronic diseases and associated risk factors [4]. Additionally, resistance training effectively increases muscle strength (1-repetition maximum) and size (muscle cross-sectional area), which also reduces the risk of age-related chronic diseases [5]. However, only 55.4% of adults aged 18–64 years old undertake 150 min of exercise per week [6]. Moreover, the magnitude of cardiorespiratory fitness adaptations from conventional exercise interventions is not consistent between individuals. Therefore, efforts have been directed to multi-omic approaches to characterize and elucidate the molecular mechanisms which underpin exercise adaptations [7,8]. This brief review aims to describe the literature reporting exercise response, outline internal and external factors involved and highlight potential areas for future research.

## 2. Interindividual Variance

There is substantial variance in the magnitude of adaptations in muscle strength, size, and cardiorespiratory fitness after several weeks of either resistance or endurance training. The HERITAGE family study [9] showed a wide range in cardiorespiratory fitness via maximal oxygen uptake ($\dot{V}O_{2}$_max_) changes after 20 weeks of endurance training in 481 adults. They observed a mean improvement in $\dot{V}O_{2}$_max_ of 0.4 L·min^−1^ (19%) with a standard deviation of 0.2 L·min^−1^; 7% of subjects experienced improvements of over 1 L·min^−1^, but 7% changed less than 0.1 L·min^−1^. Similarly, there is large variability in muscle size and strength changes in men and women after resistance training [10]. Changes in male (n = 243) and female (n = 342) muscle size and strength in the elbow flexors of the non-dominant arm were determined after 12 weeks of progressive unilateral resistance training. The mean change in muscle cross-sectional area was 20.4% for men and 17.9% for women, however, the size changes ranged from −2 to +59% (−0.4 to +13.6 cm^2^). The mean change in 1 repetition maximum for men and women was 39.8% and 64.1% respectively, with strength gains ranging from 0 to 250% (0 to +10.2 kg). This variance of exercise response has been observed in a multitude of studies, which has led to collective research efforts to uncover the determinants of exercise training response [7,11,12].

## 3. Classifying Exercise Response

There is considerable variance in the approach to research design to identify and define individual responsiveness [11]. A recent consensus statement has discussed the strength and limitations of different methods and definitions for classifying exercise response, and is recommended reading for further detail [11]. In brief, individual fitness changes must exceed random error (measurement error and day-to-day variability) to be considered a true response to the intervention [11]. Further, previous research has used arbitrary thresholds (e.g., group percentile, units of standard deviations) to categorise high, low or non-response [11]. Here we simply consider technical error (reported as the coefficient of variance (CV)), as the threshold to delineate between response and non-response.

The exercise training “non-responder” has become a topic of considerable research interest. The incidence of insignificant cardio-respiratory fitness improvement to standard endurance training has been reported to be as high as 40% [13]. Retrospective analysis of data published by Stratton and colleagues revealed that despite a statistical significant (*p* < 0.05) improvement in absolute $\dot{V}O_{2}$_max_ of 7% after 6 weeks of endurance training, over 50% (n = 21/39) of participants could be re-classified as “non-responders” when improvements exceeding the $\dot{V}O_{2}$_max_ CV of 4% were considered a genuine improvement [14].

However, the growing consensus is that “non-responders” to training programs are based more on anecdotal than experimental evidence, with several investigations showing that the incidence of non-responders is abolished if training intensity is higher [15,16], training frequency is increased [17] and/or the training mode switched from endurance to resistance training [18]. Additionally, extending the length of the training program reduces the incidence of non-responders to training [19]. In their 2019 review, Pickering and Kiely confirm that global responders to exercise are unlikely to exist and encourage “health professionals to create more nuanced, efficacious, and individually-focused exercise prescriptions designed to circumvent and overcome apparent non-responsiveness” [20].

We agree with the recommendations of Pickering and Kiely and propose that phrases such as “non”, “low”, or “poor” responders to endurance training be replaced with an alternate term. These terms imply that the magnitude of potential physiological response to training is less than in “high” responders. Strong evidence exists that the magnitude of cardio-respiratory change is similar between “low” and “high” responders if the training stimulus is sufficient. Montero & Lundby indicate that the magnitude of improvement in $\dot{V}O_{2}$_max_ in initially low responders matches that of high responders when training frequency is increased [17]. Consequently, we propose a term such as “stubborn” responders to describe individuals who do not initially respond positively to endurance training. Additionally, reporting on a range of variables to differentiate exercise responses should also be considered. While some individuals may not demonstrate large positive changes in $\dot{V}O_{2}$_max_, they may experience a range of other performance or health-related changes [21,22].

## 4. Age and Gender

Age does not appear to mitigate an individual’s ability to improve their cardiorespiratory fitness. Relative improvements to high-intensity interval training (HIIT) in $\dot{V}O_{2}$_peak_ have been observed in young (~28%) and old adults (~17%) [23]. Evidence suggests improvement in $\dot{V}O_{2}$_max_ with endurance training across age-groups is mainly due to increases in maximal cardiac output, rather than peripheral adaptations such as arteriovenous exchange [24]. Interestingly, a recent systematic review and meta-analyses presented evidence of sex-dependent adaptations of the left ventricle following endurance training [25]. Left ventricular end-diastolic volume and stroke volume are enhanced more in males compared to females, despite similar left ventricle hypertrophy [25].

Contrastingly, an individual’s ability to increase muscle mass with resistance training significantly decreases with age [26,27,28]. Ageing is associated with ‘anabolic resistance’, characterised by reduced activation of the mTORC1 pathway [29] and chronically reduced muscle protein synthesis [30]. Moreover, the lack of muscle quality associated with ageing may be an underlying factor in diminishing strength gains [23,31]. In addition, increases in fat-free mass and muscle cross-sectional area are greater in males compared to females [10,23], though changes in relative muscle strength are similar between males and females [10].

Whilst benefits of exercise have been observed across populations of different age and gender, more research is required to investigate and report interindividual variance within elderly populations [32] and genders using multiple exercise response variables.

## 5. Molecular Predictors of Exercise Response

### 5.1. Genetics

The precise effect of genetics on training responsiveness is a topic much-speculated upon. A recent systematic review highlighted the significant genetic influence on the variance of phenotype response specific to training type [33]. The analysis demonstrated that genetic variability contributed 44%, 72% and 10% of adaptation differences in aerobic, strength and power phenotypes, respectively [33]. However, the findings from the Studies of Twin Responses to Understand Exercise as Therapy (STRUETH) study suggest genetics may not play as substantive a role as first thought [18]. The STRUETH study observed training responses in monozygotic and dizygotic twins following three months of resistance and endurance training in a randomized cross-over design, separated by a three-month washout period. Interestingly, the study indicated that even participants with identical genetic make-up exhibited individual responses to the same exercise modalities, and stubborn responders to one training type (e.g., endurance) could be ‘rescued’ by another training type (e.g., resistance, or vice versa) [18]. This highlights the effects of exercise modalities and other exogenous factors (e.g., environment, lifestyle) that contribute to optimising individual response. Further research is required to determine if the incidence of stubborn responders to resistance training identified previously [10] can be ameliorated by altering dietary strategies, the type and speed of contractions, and/or the frequency, volume and intensity of training load. Stubborn training responders may simply need a different stimulus to be “jumpstarted” into action by finding the exercise stimulus that works for them.

### 5.2. Epigenetics & Gene Expression

Epigenetics refers to processes that alter gene expression without changing the DNA sequence and lead to modifications that can be transmitted to daughter cells. Exercise can cause epigenetic modifications and affect downstream metabolic adaptations [34]. The major epigenetic mechanisms are DNA methylation, histone modification, and regulation of noncoding RNA-associated genes [35]. Gene expression can partly explain the heterogenicity of $\dot{V}O_{2}$_max_ response to endurance training. Timmons and colleagues identified that the baseline expression of 29 pre-training skeletal muscle RNA transcripts were associated with $\dot{V}O_{2}$_max_ training response in subjects, who completed a 6-week training program consisting of four 45-min cycling sessions per week at approximately 70% of the pretraining $\dot{V}O_{2}$_max_ [36]. The positive correlation to improvements in $\dot{V}O_{2}$_max_ and predictive value of the 29 transcripts were confirmed in an independent training project of 17 participants who trained on a cycle ergometer five times per week for 12-weeks [36]. Basal gene expression of the predictor genes was not affected by training and therefore demonstrated their capacity to predict training response from baseline [36]. However, these results have not been replicated and require further research [8].

MicroRNAs (miRNA) and other noncoding RNA molecules are essential for muscle development and function [37]. There are several muscle diseased states associated with abnormal miRNA function [37], therefore, given that muscle development and function are influenced by miRNAs, there are likely candidate miRNAs that influence muscular adaptation to resistance and endurance training [38]. Furthermore, miRNAs that regulate protein synthesis also have the potential to partly discern the heterogenicity in muscle mass improvements from resistance training. In a study by Davidsen and colleagues, vastus lateralis biopsies were obtained from the highest and lowest ~20% of responders from 56 men who completed a 5-day per week resistance training program for 12-weeks [39]. They reported four MicroRNAs were differentially expressed between high and low responders. miR-378, miR-29a, and miR-26a were downregulated in low responders and unchanged in high responders, while miR-451 was upregulated only in low responders [39]. The resistance training-induced change in miR-378 abundance was positively associated with muscle mass increases [39]. Only 21 microRNAs were investigated in Davidsen’s project, but the Human genome currently encodes 2600 mature microRNA [40]. Consequently, there is scope to investigate the potential role of more microRNA candidates in facilitating resistance and endurance training adaptations.

### 5.3. Metabolomics

Metabolomics refers to “the quantitative measurement of the dynamic multiparametric metabolic response of living systems to pathophysiological stimuli or genetic modification” [41]. It involves the measurement of endogenous and exogenous metabolites involved in the myriad of metabolic reactions that occur in the human body, found in biofluid (plasma, saliva, breath, etc.) [42,43]. Metabolomic studies have investigated the reactions of the human metabolism in response to exercise to identify metabolites related to exercise mode, duration, and intensity [43,44]. Furthermore, a recent metabolomic study using mice identified a time-of-day-dependent impact of exercise on skeletal muscle metabolism [45], highlighting the possibilities for metabolomic investigations into uncovering the potential of exercise and metabolism. However, whilst the current evidence has characterized metabolite concentrations to exercise [43], further research is required to verify the reliability of measurements before individual metabolites or metabolic systems can be used to differentiate exercise responsiveness [46].

A potential alternative to monitor training response may be found in the breath. Exhaled volatile organic compounds are substances derived from exogenous or from endogenous metabolism, measured in parts-per-million, parts-per-billion or parts-per-trillion [47]. Metabolites that are produced within the body, pass from the blood into the lungs for exhalation, and can be detected by gas-chromatography mass-spectrometry [48]. Research in the area has predominantly focused on detection of disease (e.g., lung cancer, diabetes, asthma) and the effects of environmental factors (e.g., chlorine or petrochemicals) [49]. Previous studies have also observed this. Exhaled volatile organic compounds change in response to low-intense exercise; however, this appears to lack application in exercise and sport [49]. A recent pilot study aimed to investigate the impact of maximal exercise to exhaustion on exhaled volatile organic compounds [50]. The results indicated baseline samples of acetone and isoprene are reduced in participants with higher absolute $\dot{V}O_{2}$_max_ scores, and demonstrating dynamic isoprene concentration changes in response to exercise [50].

### 5.4. Cell-Free DNA and DNA Damage

Circulating cell-free DNA (cfDNA) refers to the extracellular strands of DNA observed in blood plasma or serum [51]. Concentrations of cfDNA increase due to exercise-induced physiological stress as well as inflammatory and immunological responses to injury or disease [20,52,53]. There are several potential mechanisms for the release of cfDNA, including programmed cell death (apoptosis), necrosis, NETosis, pyroptosis, active secretion, or impaired clearance [53]. The levels of cfDNA concentration in the blood may range between 0–5 and >1000 ng/mL in cancer patients, compared to 0–100 ng/mL in healthy people, and it has been considered a potential measure for monitoring disease status [52].

Interestingly, cfDNA has recently been investigated for its association with exercise performance [54], exercise-induced stress, and muscle damage [55]. Cell-free DNA increases have been observed following acute bouts of endurance and resistance exercise [51]. Time-course changes of cfDNA typically peak between 0–2 h after exercise and gradually return to baseline after 24 h, and early evidence suggests exercise-induced increases in cfDNA are dose-dependent, similar to the inflammatory response [51]. Andreatta et al. [55] compared acute bouts of light (40% 1-RM) and heavy (80% 1-RM) resistance exercise and monitored cfDNA levels with functional muscle capacity up to 48 h after exercise. The concentration of cfDNA only increased following the heavy session and was associated with squat jump performance decrements up to 48 h after exercise [55]. This highlights cfDNA’s potential as a measure of training status and adaptation, which has been suggested previously [56,57].

The effect of endurance exercise intensity on cfDNA has not been fully elucidated, as the majority of studies have completed endurance exercise to exhaustion [54,58,59,60]. Despite this, a recent systematic review and meta-analyses found DNA damage is significantly increased after higher-intensity exercise (>75% $\dot{V}O_{2}$_max_) and was not significantly higher after long-distance exercise (>42 km) [57]. The authors suggest that this may be due to analytical factors, or that DNA damage repair and removal may occur during prolonged endurance exercise [57].

It is uncertain that cfDNA is detrimental to human health or performance. More research is required to characterize cfDNA fluctuations and their association with long-term health, as well as following different exercise intensity, volume, duration, or modes. Furthermore, clarifying the reliability of cfDNA responses to the same exercise bout will allow a basic understanding of its significance during training load monitoring.

### 5.5. Exerkines

Recently, the links between exercise and “exerkines” have been speculated as possible mediators of exercise adaptation [61,62]. Exercise training adaptation is a consequence of the accumulative application of repeated exercise stimuli. There is growing interest in that progressive adaptation to endurance and resistance training is mediated by the simultaneous integrative effect of several organs in response to exercise [61,62]. The act of exercise releases hundreds of biologically active compounds into the blood. These compounds called exerkines are released from many of the body’s organs, tissues, and cells and exert auto-, para or endocrine effects. Exerkines are released from skeletal muscle (myokines), the heart (cardiokines), liver (hepatokines), white adipose tissue (adipokines), brown adipose tissue (baptokines) and neurons (neurokines) [62].

Many of these exerkines have been shown in isolated muscle cells and rat models to potentially regulate muscle protein synthesis (Myostatin, Follistatin, IL6, IL8, Il7, IL15, Decorin, VEGF, Leukemia Inhibitory Factor), vascularization (VEGF, IL8), mitochondrial biogenesis (Apelin), muscle repair and remodelling and heart remodelling (Brain Natriuretic Peptide & Musculin) and neuroprotection in the hippocampus (Brain-Derived Neurotrophic Factor) [61].

However, the relationships between exerkines and muscle strength, size and cardiorespiratory adaptations in response to training have yet to be fully elucidated and warrant further investigation. Furthermore, we hypothesize that candidate biomarker response to an exercise bout offers more insight than assessment in a rested state, as the individual’s unique physiological response to exercise is likely more permissive in facilitating adaptation than a static non-stimulated biomarker [63].

## 6. Effects of Exercise Variables

Non-response to endurance training appears to be abolished by increasing exercise dosage (e.g., duration, volume, time or intensity, etc.) [16,17]. Montero and Lundby [17] compared the prevalence of cardiorespiratory non-response among 78 healthy adults undertaking 60, 120, 180, 240, or 300-min of endurance training per week for 6 weeks. Participants were divided into five groups (1–5) that completed one, two, three, four, or five 60-min exercise sessions. Non-response was defined as any change in cardiorespiratory fitness determined by maximal incremental exercise power output, within the typical error of measurement (±3.96%). Importantly, non-response was negatively associated with exercise dose in the first 6 weeks, with groups 1, 2, 3, 4, and 5, 69% (11 of 16), 40%, 29%, 0%, and 0% of individuals, respectively [17]. Interestingly, non-responders completed an additional 6 weeks of endurance training with two extra sessions per week, which eliminated all prevalence of non-response. It could be argued that it was the successive training period (e.g., additional 6 weeks) after less than 7 days washout period that contributed to the physiological adaptations. However, another study also observed the separate effects of exercise intensity and volume over a 24-week intervention period (low intensity, low volume vs. low intensity, high volume, vs. high intensity, high volume) and found 38.5% (15 of 39), 17.6% (9 of 51), and 0% (0 of 31) of participants within the respective groups were non-responders [16].

Evidence is accruing that the probability of improving cardiorespiratory fitness is enhanced if training is anchored to the individuals’ metabolic threshold rather than training to generic heart rate zones recommended by guidelines. Weatherwax and colleagues determined the incidence of $\dot{V}O_{2}$_max_ responsiveness to standardized or individually prescribed exercise based on ventilatory threshold [15]. Forty sedentary adult participants completed endurance training on three days per week for 12-weeks, with standardized exercise prescription based on a percentage of heart rate reserve (HRR) or with an individualized approach using ventilatory thresholds (VT). The standardized groups’ initial exercise intensity used a target heart rate zone at 40–45%HRR and progressed to 60–65%HRR by week 12, compared to the individualized group which started within 10 beats per minute below VT1, progressing to within 10 beats per minute above VT2 by week 12 [15]. Each group was matched for energy expenditure using kilocalories per kilogram of body mass per week. The study implemented a verification protocol to establish the site- and cohort-specific typical error (biological variability and measurement error) [13], determining that $\dot{V}O_{2}$_max_ changes need to be greater than 4.7% to be considered a true response [15]. There were no significant changes to body mass observed between groups but relative $\dot{V}O_{2}$_max_ significantly increased from 24.3 to 26.0 and 29.2 to 32.8 mL·kg^−1^·min^−1^ for the standardized and individualized groups, respectively. More interestingly, a significant difference in the number of responders was found between the groups with 100% of the individualized training group categorized as responders but only 60% in the standardized training group categorized as responders [15].

Manipulation of resistance training variables to maximize muscle strength and hypertrophy has been widely investigated [64,65,66]. While there are some crossover effects, training for muscle strength or hypertrophy requires separate loading schemes to optimise specific training adaptations [65]. This is due to increases in strength being preceded by neuromuscular changes that increase the capacity to produce force [67]. A study compared neuromuscular adaptations following 3 and 6 weeks of 80% vs. 30% 1RM resistance training to failure in the leg extensors of 26 healthy adult males (23.1 ± 4.7 years) [67]. While they observed similar increases in muscle thickness, 1-RM and maximal voluntary isometric contraction were significantly greater in the 80% 1-RM training group [67]. These echo the results of a recent meta-analysis which found the magnitude of muscle strength increases to be greater in resistance training loads above 60% 1-RM compared to resistance training below 60% 1-RM [68]. Interestingly, while high-load resistance training is necessary to maximise muscle strength, either high or low loading is capable of eliciting muscle hypertrophy so long as it is completed to failure [69]. While it may not be pragmatic in an applied setting, to reduce the interindividual variance of hypertrophic responses during resistance training studies, it is suggested to perform the exercise to volitional fatigue (failure) to ensure all participants undergo a standardized stimulus [64].

## 7. Conclusions

For the public to embrace prior determination of their disposition to improving cardiorespiratory fitness, muscle strength and size to exercise training, biomarker tests should be tolerable. The physiological adaptability screening test(s) should be minimally invasive and restricted to blood, urine, saliva, or breath samples, affordable, and the analysis should be conducted efficiently and promptly. For example, muscle biopsies provide valuable mechanistic insight into the biology of heterogenicity of training adaptation response but have limited practical relevance to the general public and clinical populations as they are invasive, require medical supervision, are expensive and require specialized analytical skills and equipment. Research should focus on candidate biomarkers of trainability that are more likely to be embraced by the community and can be performed in commercial settings (e.g., gymnasiums and exercise physiology clinics). We propose that the initial response in the metabolome and exerkine response to a resistance or endurance workout at personalised intensities before engaging in long-term training should be investigated as possible biomarkers that predict muscle strength and size or cardiorespiratory fitness trainability, respectively. The magnitude of adaptation to exercise training is far from simple. However, an investigation into the interaction of the myriad of potential facilitatory contributors to muscle and cardiorespiratory adaptations will ultimately progress the emerging recognition that exercise prescription should be personalized to optimize the effect on an individual’s athletic performance and health.

## 8. Future Directions

The field of personalized medicine is best evolved by understanding the physiological and environmental regulators of adaptation. The ultimate goal should focus on developing commercially viable tests that can predict trainability. To expand on research that has shown the relationships of gene sequence and gene expression to training adaptations, studies should investigate the relationship of exerkines, the metabolome and DNA damage to endurance and strength training adaptations after several weeks-to-months of training. In addition, training programs should be prescribed using individualised methods (e.g., anaerobic threshold, critical power) to reduce the variance of responses caused by generic prescription methods (e.g., percentage of maximum heart rate). Studies assessing exercise response in skeletal muscle hypertrophy may also benefit by standardising the exercise stimulus by completing the exercise to volitional fatigue (failure). This may be verified by measurement of mean concentric velocity and velocity loss throughout the working set (e.g., velocity-based training), combined with a rating of perceived exertion.

The diagnostic value of biomarkers to predict trainability should be assessed in basal/rested state and in acute response to an initial endurance or resistance training session. We hypothesize that the biomarker response to an exercise bout is more likely to reflect its capacity to induce/reflect the physiological disturbances that evoke adaptation to exercise [63]. To achieve this, it is crucial that the reliability of these candidate biomarkers are established and the test-retest error reported as coefficient of variation and standard error before investigating their relationship to training. For a biomarker to have clinical diagnostic value its relationship to the intensity and duration of endurance and strength training should be established. The biomarker should possess high construct validity and the capability to determine between light and intense training sessions. It would also be of value to determine if contraction type (eccentric v concentric) effects the biomarkers differently. In addition, the utility of the biomarkers for understanding individual responsiveness should be characterized in healthy populations before being used in assessing individual responsiveness in diseased populations.

All studies should report individual changes in the dependent variable measured (e.g., $\dot{V}O_{2}$_max_, 1-RM, CSA) and not just the group mean of the dependent variable. Presentation of individual responses in waterfall style graphs enables the readers to gauge the relative proportion of low and high responders to training. The accompanying waterfall graph should incorporate a line that delineates the measurement or critical error of the dependent variable to enable the reader to discern a genuine intervention effect from the test’s “noise”.

Ultimately for a biomarker of trainability to have practical relevance, it needs to be palatable to the general population. Minimally non-invasive options that can be assessed relatively cheaply and quickly would be more appealing and maximize the percentage of the population that would embrace a physiological test of trainability. Breath analysis of volatile organic compounds has potential to be a non-invasive measure which monitors training status and trainability. However, more research is needed to characterise volatile organic compounds following endurance or resistance exercise sessions. 

## Data Availability

Not applicable.
